# Therapeutic Potential of Chitosan-Based and Related Nanocomposite Systems in Wound Management: A Review

**DOI:** 10.3390/ijms262311748

**Published:** 2025-12-04

**Authors:** Beata Bielska, Katarzyna Miłowska

**Affiliations:** 1 Department of General Biophysics, Faculty of Biology and Environmental Protection, University of Lodz, 90-236 Lodz, Poland; katarzyna.milowska@biol.uni.lodz.pl; 2 Doctoral School of Exact and Natural Sciences, University of Lodz, 90-237 Lodz, Poland

**Keywords:** chitosan, polyphenols, chronic wounds, biomaterials, wound healing, regenerative medicine

## Abstract

Chronic wounds, particularly those associated with diabetes, persist as a significant clinical challenge due to prolonged or incomplete healing, elevated infection rates, and the ensuing risk of lower-limb amputation. This review summarises recent advances in biomaterials for wound healing, focusing on chitosan-based systems modified with metal nanoparticles and polyphenols. The text emphasises the pivotal function of nanotechnology in facilitating targeted delivery and synergistic bioactivity. The present study places particular emphasis on the synergistic use of chitosan and polyphenols in drug delivery systems and next-generation wound dressings. This combination successfully overcomes the key limitations of their individual use, such as the poor solubility of polyphenols and the limited stability of chitosan. The encapsulation of polyphenols within the nanostructures of chitosan is a process enabled by nanotechnology. This process has been shown to enhance the bioavailability of the polyphenols, to allow for controlled release, and to improve their biological performance. This review methodically synthesises the extant experimental evidence demonstrating that these multifunctional systems exhibit regenerative, antioxidant, and antimicrobial properties that may support selected biological processes relevant to wound repair. The promotion of angiogenesis, fibroblast growth, and epithelial regeneration is accompanied by a reduction in infection-related complications. Whilst the majority of the studies under review are of a preclinical nature, the body of evidence suggests that further enhancement and quantitative evaluation of these systems has the potential to pave the way for clinically relevant therapies for chronic and diabetic wounds.

## 1. Introduction

The skin, an essential organ that regulates the body’s balance, forms the first line of defence against infection. Disruption of the functional and anatomical integrity of this barrier can lead to fluid loss, such as water or electrolytes. A breach in the skin’s integrity has been demonstrated to induce pathological disturbances that delay the body’s return to homeostasis. The disruption of the continuity of the skin and underlying tissues resulting from a wound creates an entry point for infections and disturbs internal balance [1,2]. The introduction of pathogens into the affected area has been demonstrated to result in a delay in wound healing and the restoration of normal physiological conditions [3]. In the case of chronic wounds, the healing process is characterized by a duration exceeding three months and is typified by a protracted and persistent inflammatory phase that impedes the capacity of dermal and epidermal cells to respond to chemical signals [4]. It is estimated that 6.5 million patients are affected by chronic wounds with the annual cost of treatment amounting to approximately $25 billion [4]. Chronic wounds are a significant economic burden on healthcare systems, due to higher costs associated with dressing changes, hospitalisations, and nursing care. The most recent 2025 report on the global wound dressing market, indicates that the market was valued at $9.39 billion in 2024, and it is projected to grow to $16.94 billion by 2032 [5,6].

The predominant clinical challenge associated with chronic wounds is their persistent inability to achieve timely, complete, and durable healing. Notwithstanding the advances that have been made in the field of wound management, contemporary therapeutic outcomes remain inadequate. Patients frequently encounter markedly protracted healing trajectories, persistent infection-related complications and difficulties in maintaining sustained wound closure. These factors collectively impede the restoration of functional tissue integrity. Furthermore, the presence of comorbidities, such as diabetes, has been demonstrated to exacerbate the risk of delayed wound healing [7]. In the context of diabetic wounds, these difficulties are further amplified by metabolic dysregulation and impaired immune responses, contributing to a markedly higher likelihood of serious complications. Achieving clinically acceptable outcomes, therefore, necessitates demonstrable improvements in healing dynamics, enhanced management of infection-associated complications, and consistent progression towards stable and sustained tissue regeneration.

Globally the prevalence of diabetes has reached epidemic proportions, with an estimated 830 million individuals affected worldwide. Elevated blood glucose levels have been demonstrated to induce significant cardiac dysfunction, in addition to damage to nerves and blood vessels [8,9]. It is imperative to note that even minor cuts or bruises in diabetic patients pose a high risk of dangerous complications. The initiation of repair processes in diabetic wounds poses a significant clinical challenge due to a number of factors. Firstly, there is an increased infection risk. Secondly, there is a reduced fibroblast migration and proliferation. Thirdly, there is limited angiogenesis. Finally, there is decreased collagen production caused by prolonged hyperglycaemia. Wounds in patients with diabetes mellitus, due to impaired capillary formation (angiogenesis)—the process responsible for delivering nutrients and oxygen to the damaged area—exhibit a prolonged healing time, thereby creating a favourable environment favorable for bacterial colonization [10]. Consequently, meta-analytical evidence suggests that approximately 31% of patients with diabetic foot ulcers undergo lower limb amputation, as clinicians attempt to manage symptoms and prevent the progression of infection in the absence of therapies that adequately support tissue regeneration [11]. The challenging conditions under discussion can be attributed to excessive oxidative stress, which has been demonstrated to impede angiogenic responses and result in endothelial dysfunction. The absence of an environment conducive to endogenous growth factors, cells, tissues, or exogenous treatments hinders the development of efficient therapies, exposing diabetic patients to further health complications [12]. There is an imperative need for a sophisticated wound dressing capable of managing and eradicating infections, sustaining optimal moisture levels, safeguarding the wound, reduces inflammation, facilitating fibroblast proliferation, and promotes angiogenesis. This is paramount for ensuring the delivery of nutrients to the compromised tissue. In addition, the dressing should be biodegradable or capable of facile removal following the healing process. This is in order to minimise the risk of secondary skin injuries and ensure complete restoration of the skin structure [13].

**Figure 1:**
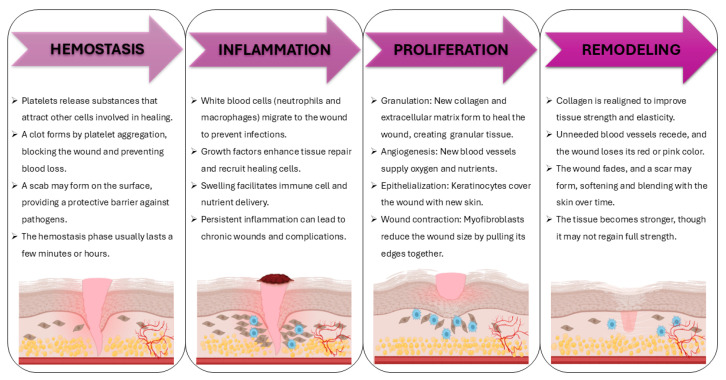
Phases of cutaneous wound healing: Hemostasis, Inflammation, Proliferation, and Remodelling. Adapted from [16].

The process of wound healing is defined as the series of events that lead to the closure of a wound and the restoration of disrupted cellular connections. This process is achieved through the growth and regeneration of tissue, which is stimulated by biochemical and cellular mechanisms [14]. The restoration of skin integrity is achieved through a coordinated cascade of complex and dynamic processes, which can be divided into four key stages: hemostasis, inflammation, proliferation, and remodelling of the epithelium and tissues (Figure 1) [7,15].

The process of skin repair is initiated by coagulation, which is the formation of a platelet plug that stops bleeding. This stage occurs immediately after skin injury. This is followed by an inflammatory response, which lasts approximately three days and during which growth factors are released from platelets and damaged keratinocytes. The elimination of microorganisms by inflammatory cells is achieved through the production of reactive oxygen species and proteases, which combat pathogens and remove dead cells and debris from the wound bed. In the subsequent phase, a range of cell types proliferate and migrate, concurrently secreting growth factors, cytokines, and chemokines. It has been observed that keratinocytes migrate into the damaged dermis, while the fibrin matrix is replaced by fibroblasts, forming what is known as granulation tissue. Concurrently, fibroblasts secrete angiogenic growth factors, including vascular endothelial growth factor (VEGF), thereby facilitating re-epithelialization. In the final stage, remodelling occurs, characterised by protein synthesis, wound contraction, and connective tissue reconstruction, ultimately resulting in scar formation. Delays in skin regeneration have been shown to encourage the use of dressings that promptly aid wound closure and the formation of new tissue [17].

**Figure 2:**
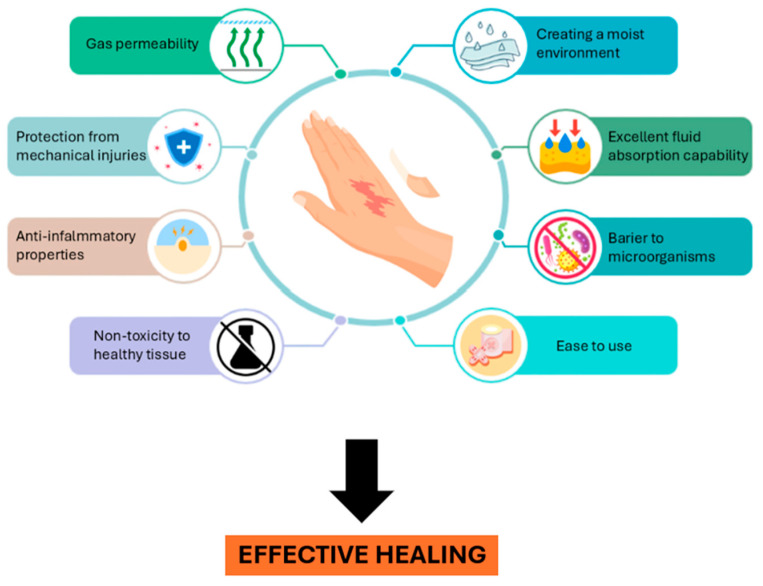
Key physicochemical and biological features of an ideal wound dressing that support effective healing.

Despite the continuous advancements witnessed in the domain of wound management, contemporary therapeutic interventions have yet to guarantee efficacious healing outcomes for chronic and diabetic wounds. This persistent challenge can be attributed to the presence of inflammation, bacterial resistance, and impaired tissue regeneration. Consequently, there is an increasing demand for multifunctional biomaterials that are capable of addressing these specific clinical challenges through combined antimicrobial, antioxidant, and regenerative mechanisms. The application of suitable therapeutic dressings has been shown to help prevent complications that hinder skin regeneration and delay patient recovery (Figure 2). It is a well-documented fact that commonly used medical materials have the capacity to dehydrate the wound, adhere excessively to the wound bed, and create an anaerobic environment conducive to microbial growth. It has been demonstrated that alterations in patients’ attire can induce discomfort and pain, thereby impeding the healing process [18]. Despite the advances witnessed in the design of modern wound care materials, a considerable number of currently employed dressings still fail to satisfy the minimal requirements necessary to achieve clinically acceptable outcomes in patients with chronic wounds. In diabetic wounds, the most critical clinical challenge remains the persistently slow and incomplete healing, which contributes to recurrent infections and disruptions in key regenerative processes such as angiogenesis and fibroblast proliferation. It is imperative that wound dressings possess the capacity to fulfil multiple functions, including the maintenance of a moist environment, the regulation of fluid absorption, the facilitation of gas exchange, and the provision of mechanical protection. In addition to these fundamental properties, it is essential that wound dressings demonstrate the ability to reduce bacterial burden, modulate inflammation, and support tissue regeneration, thereby enhancing clinical outcomes in a substantial manner [7,8,9]. Throughout the subsequent sections, the review also emphasises the relevance of wound aetiology and healing stage, discussing how the applied composites interact with and influence the wound-healing process in these distinct clinical contexts. The efficacy of a particular dressing is contingent upon the nature of the wound, its historical context, prior therapeutic interventions, and the frequency of dressing changes [4]. Despite the growing number of studies on chitosan-based biomaterials and polyphenol applications in wound therapy, a systematic mechanistic overview integrating both systems remains lacking. This review seeks to deliver a comprehensive and critical overview of chitosan-based composites incorporating polyphenols and metal nanoparticles, while concurrently comparing their performance with other polymeric systems employed in wound healing. The originality of this work lies in its integrative approach, which combines the structural and functional versatility of chitosan with recent advancements in nanotechnology to enhance antimicrobial, antioxidant, and regenerative outcomes in the treatment of chronic wounds.

Although a considerable number of experimental studies have demonstrated the beneficial biological effects of chitosan–polyphenol composites, their translation into clinically applicable therapies remains limited. The present review aims to summarise the current findings, primarily obtained from in vitro and in vivo studies, and to evaluate the potential modifications required to translate these results into clinical applications. The study goes on to assess which materials and structural modifications appear most promising for clinical implementation. The present review has been conceived with a view to establishing a connection between material design and biological mechanisms. To this end, it will highlight the synergistic role of chitosan–polyphenol nanocomposites in the process of wound healing.

## 2. Nanostructures

**Figure 3:**
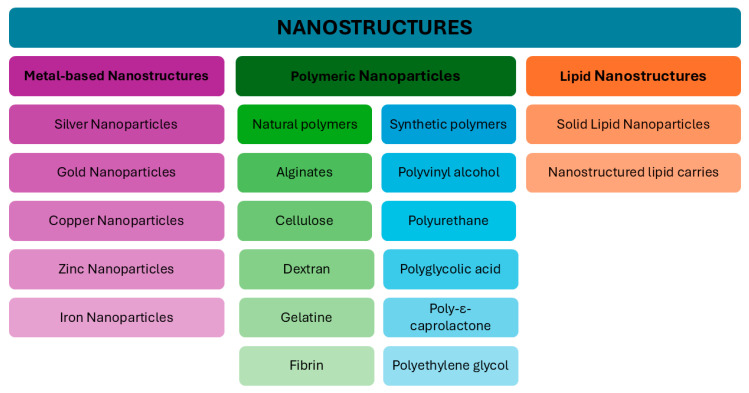
The classification of selected nanostructures that have been tested and used in wound healing, along with selected representatives of group.

The field of nanotechnology has also attempted to address the challenge of wound healing by developing advanced materials that promote tissue regeneration, prevent infections, and enhance the delivery of therapeutic agents directly to the wound site [19]. The utilisation of nanostructure-based delivery systems has emerged as a promising avenue for the development of innovative dressings a field that is characterised by their numerous advantageous properties [7]. Nanostructures used in wound healing can be categorised into three primary types: inorganic/metallic, lipid-based, and polymeric (Figure 3) [7]. Inorganic nanoparticles, including silver, gold, and zinc oxide, have been demonstrated to possess potent antimicrobial properties, promote angiogenesis, and hinder infection at the wound site, making them widely used in dressings. Lipid-based nanoparticles are biocompatible and are readily absorbed by the body, rendering them optimal carriers for drugs such as antibiotics or growth factors that promote tissue regeneration. Polymeric nanostructures, composed of materials such as chitosan or alginate, facilitate regulated drug release, enhance moisture levels at the wound site, and accelerate regenerative processes. These structures are characterised by biodegradability and versatility in medical applications. The delivery of drugs and growth factors directly to the wound site by nanoparticles allows for precise control of their release and protection of active ingredients from degradation [7]. Furthermore, the use of nanoparticles has been demonstrated to enhance the bioavailability of therapeutic agents and facilitate better penetration through skin layers, which is critical for the successful treatment of chronic diabetic wounds. It has been demonstrated that biocompatible nanoparticles (NPs), such as zinc oxide, gold, and silver, exhibit both bacteriostatic and bactericidal properties while maintaining low in vivo toxicity [3,20]. By reducing material size to the nanoscale, these particles provide a larger surface area and altered physicochemical properties, accelerating the regeneration process [21].

## 3. Polymeric Nanomaterials

Polymers are defined as substances composed of long chains of monomers. It is evident that both synthetic and natural polymers demonstrate advantageous characteristics that facilitate wound healing. These polymers offer biocompatibility, adaptability for a range of applications, and ease production, thereby providing a foundation for their potential utilisation in wound healing processes. Synthetic polymers, including polyurethanes and natural polymers such as gelatin, fibrin, chitosan, and cellulose, have key roles in tissue engineering, drug delivery systems, and wound dressing development. Polymers are frequently utilised in the form of hydrogels, which are water-retaining materials with high biocompatibility. The utilisation of hydrogels in the production of bandages and wound dressings is a viable proposition.

Their capacity to adapt to the configuration of wounds renders them more versatile than conventional scaffolds. Polymer-based hydrogels are a promising platform for the localised and sustained delivery of growth factors to wound sites. In a study conducted by Lin et al. ([22]), polyurethane (PU)/hydrogel composites incorporating fibroblast growth factor-2 (FGF-2) were investigated for their wound healing efficacy in an in vivo Sprague Dawley rat model. Full-thickness excisional wounds were surgically induced on the dorsal skin in order to replicate clinically relevant dermal injuries. The incorporation of FGF-2 into the hydrogel matrix led to a substantial enhancement in the rate of wound closure in comparison with control groups. This was accompanied by an increase in fibroblast proliferation and enhanced neovascularization. These results underscore the translational potential of FGF-2-loaded hydrogel systems, particularly in the context of chronic wounds where impaired angiogenesis and reduced fibroblast activity present substantial barriers to tissue regeneration [22,23].

### 3.1. Chitosan

The wide range of biocompatible polymers has prompted research into suitable compounds for use in therapeutic dressings. Various substances have been analysed and a key polymer, chitosan, has been selected. This has become the basis for creating medical materials in various forms, including films, sponges, fibres and hydrogels. Over time, combinations of chitosan with other compounds that exhibit excellent medical properties have been synthesised to produce materials with optimal therapeutic specifications [24].

This natural biopolymer consists of N-acetyl-D-glucosamine and D-glucosamine units featuring active cationic amino groups and hydroxyl groups. These give the polymer unique polyelectrolyte properties, chelating abilities, and film-forming potential. Additionally, the positive cationic charge of chitosan (arising from protonated amino groups at acidic pH) facilitates electrostatic interactions with negatively charged bacterial membranes [25]. From another perspective, enhanced NH_2_ groups increase chitosan’s ability to form hydrogen bonds with other polymers, such as cellulose, thereby improving its mechanical and physicochemical properties while preserving its unique attributes [26]. This organic compound is characterised by valuable biological properties, making it an ideal component of wound dressing materials. Chitosan-based materials have been approved by the U.S. Food and Drug Administration (FDA) for use in bandages and other hemostatic applications [27]. This chitin derivative exhibits antibacterial properties and is biocompatible and biodegradable. It also has antioxidant, hemostatic, and immune-stimulating effects [3,6].

Chitosan has a structure similar to glycosaminoglycans (GAGs) found in the extracellular matrix (ECM). These GAGs play a role in regulating the activity of macrophages, fibroblasts, and endothelial cells, thereby supporting the formation of granulation tissue [28]. During the hemostatic phase, chitosan can bind to erythrocytes, facilitating rapid blood clotting [24]. It has also been suggested that interactions between positively charged chitosan molecules and negatively charged bacterial cell membranes disrupt membrane integrity [28]. Although chitosan exhibits antibacterial effects against a wide range of microorganisms, its efficacy is limited to acidic conditions, above pH 6.5, it loses its cationic character and, consequently, its antibacterial properties. The mechanism of bacterial growth inhibition likely involves the positively charged polymer binding to anionic components such as N-acetylmuramic acid, sialic acid, and neuraminic acid, which are present on the cell surface and affect cell wall permeability. Chitosan can also form an impermeable layer around the bacterial cell, preventing the transport of essential solutes. Furthermore, low molecular weight chitosan molecules can penetrate bacterial cell walls, bind to DNA, and inhibit mRNA synthesis and DNA transcription [25]. Chitosan supports the formation and organisation of granulation tissue by modulating the functions of inflammatory cells such as macrophages, fibroblasts, keratinocytes, and endothelial cells [24]. Highly deacetylated chitosan (89%) has been shown to strongly stimulate fibroblast proliferation [29]. These benefits have prompted researchers to use chitin derivative-based materials as therapeutic dressings in wound healing. However, despite its numerous advantages, the high molecular weight and viscosity of chitosan make it insoluble in most organic solvents and non-acidic aqueous media, restricting its applications. To overcome these challenges, the functional groups of chitin derivatives are modified.

Carboxymethyl chitosan (CMCS) is a water-soluble derivative of chitosan containing carboxyl groups, as well as the native amino and hydroxyl functionalities. This modification significantly improves its solubility in neutral and alkaline environments, unlike native chitosan, which is only soluble in acidic conditions [30]. The relatively simple synthesis of CMCS, combined with its excellent biocompatibility, has attracted growing interest in its biomedical applications, particularly in the context of wound healing. In a recent in vivo study, Kłosiński et al. (2023) demonstrated that CMCS-based hydrogels significantly accelerated the healing of full-thickness skin wounds in rats, particularly in models of difficult-to-heal wounds [31]. Histopathological analyses revealed reduced inflammatory cell infiltration, enhanced early collagen deposition, and improved re-epithelialisation. Furthermore, the hydrogels were non-cytotoxic, underscoring their therapeutic potential in regenerative wound care. More advanced CMCS-based systems—such as hydrogels crosslinked with polyacrylic acid and loaded with ultrasmall gold nanoparticles (PAA-CMCS-Au/UsAuNPs), have exhibited potent antibacterial and antioxidant properties. These materials have been shown to strongly inhibit pathogens such as *Staphylococcus aureus* and *Escherichia coli* by disrupting bacterial membranes and interfering with metabolic pathways [32]. In another study, Chang et al. ([12]) developed a CMCS-heparin hydrogel for use as a drug delivery system. This demonstrated favourable effects on wound healing in diabetic models [12].

From a biodegradation standpoint, CMCS is partially susceptible to enzymatic cleavage by lysozyme. However, the incorporation of carboxymethyl groups alters both the rate and the mechanism of degradation, which vary depending on the degree and site of substitution. This results in a slower and less predictable enzymatic breakdown. While prolonged material persistence may be advantageous for sustained therapeutic action at the wound site, concerns are raised about the fate of residual polymer fragments—particularly in composite systems containing metallic nanoparticles or synthetic cross-linkers [31,32].

Despite promising outcomes in preclinical models, several translational barriers must still be overcome. These include the absence of standardised synthesis protocols, limited regulatory classification, and a lack of robust clinical data regarding long-term safety, biodegradation products, and biocompatibility. Addressing these challenges is essential if the clinical potential of CMCS-based biomaterials for advanced wound dressings and drug delivery applications is to be realised.

Chitosan derivatives are also used in hemostatic sponges (MACS), which rapidly stop hemorrhages that are resistant to spontaneous coagulation. These sponges demonstrate rapid water and blood absorption capabilities, while maintaining structural integrity. Consequently, they are able to apply suitable pressure to wounds, thereby effectively managing hemorrhaging. Compared to conventional clinical materials such as gauze, gelatin sponges, or CELOX™ gauze, chitosan-based sponges demonstrate superior procoagulant and hemostatic properties in rat and pig liver perforation wound models, both under normal and heparinised conditions. They also exhibit antibacterial activity against *S. aureus* and *E. coli* and stimulate hepatic parenchymal cell infiltration, angiogenesis, and tissue integration in rat liver injury models. These sponges are particularly suitable for patients with non-compressible hemorrhages, which pose a life-threatening risk [33].

### 3.2. Other Natural Polymers

Natural polymers are derived from microbial, animal, or plant biomass. Their biodegradability, biocompatibility, and biological activity make them attractive materials for wound healing. However, their uncontrolled degradation rates can limit their use. Alginate, a linear anionic polymer consisting of β-D-mannuronic acid (M-block) and α-L-guluronic acid (G-block) units, is widely used in wound care. Alginate interacts with elastase, reduces pro-inflammatory cytokines, limits free radical production, and exhibits antimicrobial properties. In hydrogel form and combined with plant extracts or honey alginate enables the controlled release of bioactive compounds that can penetrate bacterial biofilms and disrupt the cell membrane structures of both Gram-positive and Gram-negative bacteria. Moreover, the negatively charged carboxyl groups of alginates create an electrostatic barrier that hinders bacterial adhesion to the wound surface. Alginate primarily used in hydrogel form, where its structure can be tailored for specific mechanical properties [34]. Alginate’s antiseptic properties, low toxicity, biocompatibility, excellent water absorption, and optimal water vapour transmission rates of alginate make it a promising candidate for wound healing. Gao et al. (2022) developed a 3D culture system using a collagen-microsphere hydrogel with alginate, which enhanced the viability of human umbilical cord mesenchymal stem cells and enabled their continuous release, thereby accelerating wound healing [35]. Similarly, 3D printing technology has been used to create a gelatin–alginate dressing that promotes wound closure, hair follicle regeneration, and atraumatic removal [36].

Another noteworthy polymer is cellulose, which is obtained from plants or bacteria and is a naturally abundant material. Bacterial cellulose is particularly appealing for wound healing thanks to its porous structure, which resembles skin, and its hydrophilic properties, which help to maintain an optimal moist environment at the wound site [37]. However, its lack of antibacterial activity limits its applications. To address this issue, bacterial cellulose can be modified to enhance its potential for skin regeneration. For example, combining cellulose with ε-polylysine and crosslinking with polydopamine creates a membrane with strong antibacterial properties in both in vitro and in vivo settings. This antibacterial mechanism is mainly attributed to ε-polylysine, which exerts its bactericidal effect by interacting electrostatically with negatively charged phospholipids in the bacterial cell membrane, resulting in membrane disruption, leakage of intracellular components, and subsequent cell lysis. This membrane has also demonstrated hemocompatibility and cytocompatibility in vitro and led to complete wound healing n the test group in vivo compared to open wounds in the control group [38]. Commercially available cellulose-based dressings are continuously being improved to overcome limitations, with 3D printing being a key tool for optimising wound dressing designs. Composite 3D cellulose scaffolds could potentially replace basic first-aid products such as cellulose gauze and pads. These scaffolds enhance blood clotting and promote NIH 3T3 fibroblast growth and proliferation, ultimately accelerating wound healing [39].

The limitations of polymers have driven efforts to maximise their potential. Dextran for example, is a natural polysaccharide consisting of linear chains of D-glucopyranose residues linked by α-1,6 bonds. However, it has limited use due to its slow degradation, restricted cell adhesion, and low biocompatibility. By modifying dextran with glycidyl methacrylate, researchers created a composite gel that adhered to cells and exhibited biocompatibility in vivo [40]. Dextran-based hydrogels have also shown promising in vivo results by promoting neovascularisation and skin regeneration in mice, as well as improved dermal remodelling and reinnervation in pig models [41].

Natural polymers such as gelatin and fibrin are commonly used in the development of scaffolds that promote skin repair through enhanced cell adhesion, increased angiogenesis and reduced inflammation. Studies have shown that gelatin-based scaffolds speed up wound healing in rats by providing a three-dimensional architecture that is biocompatible and biodegradable, and that resembles the key biochemical and structural features of the native extracellular matrix-. It should be emphasised that this ‘ECM-mimicking’ property does not refer to replicating its dense architecture, but rather to reproducing its biochemical composition and topographical cues. The aim is to create a porous, interconnected 3D environment that enables cells to infiltrate easily, nutrients and oxygen to diffuse, and blood vessels to grow—all of which are essential processes for successful tissue regeneration. Within this framework, gelatin scaffolds offer bioactive sites that promote fibroblast adhesion, proliferation and collagen synthesis, while their tunable porosity facilitates angiogenesis and granulation tissue development. Similarly, fibrin is widely used in wound therapy and tissue engineering due to its ability to bind a variety of bioactive molecules, supporting specific cell–matrix interactions and enhancing tissue regeneration [42]. These scaffolds provide sufficient time for neo-matrix development while being gradually resorbed by proteases [43]. Gelatin is a versatile material that can take various forms, such as hydrogels, sponges, and microspheres. Gelatin-based sponges exhibit strong antibacterial activity and cytocompatibility in vivo, which is attributed to their porous, hydrophilic structure and their ability to deliver antimicrobial agents. They inhibit bacterial colonisation, promote fibroblast proliferation and matrix formation, and support the sustained release of bioactive compounds, leading to enhanced angiogenesis, epithelialisation, and accelerated wound healing [44]. In summary, polymeric nanoparticles offer significant potential in wound healing by providing a combination of versatility, biocompatibility, and regenerative support. Advances in polymer modifications and technologies such as 3D printing continue to enhance their performance and expand their applications in regenerative medicine.

### 3.3. Synthetic Polymers

Synthetic polymers produced in laboratories can have specific structures and properties that are useful for certain applications. While they are free from contamination and stable, degrading in a controlled manner, they are mostly biologically inert [37]. Due to their lack of therapeutic benefits and limited compatibility and adhesion, synthetic polymers are often surface modified by attaching polar groups or biological components [45]. They are also frequently combined with natural polymers, which serve as biological agents to improve biocompatibility, despite their own limitations [37]. Polyvinyl alcohol (PVA) is widely used in wound-healing studies due to its high biodegradability, biocompatibility, and non-toxicity. PVA is primarily utilised in combination with natural polymers to develop composites that address its limitations [46]. For example, combining PVA with sodium alginate to form of hydrophilic, biocompatible wound-dressing films improves the chemical, physical, and biological properties of the final product [47]. Similarly, pairing PVA with bacterial cellulose addressed the weak antibacterial, mechanical, and elastic properties of PVA, enhancing interactions with various cell types [48]. In addition to improving PVA’s mechanical properties, natural polymers such as chitosan impart essential hemostatic properties that are vital for wound healing. Combining PVA with chitosan enhances mechanical durability, cell adhesion, hemocompatibility, and optimal moisture retention, thereby increasing cell migration and accelerating healing compared to conventional dressings [49]. Combining PVA with natural polymers such as sodium alginate, chitosan, gelatin, or fibrin improves water absorption, cell adhesion, proliferation, and wound healing [50].

Another synthetic polymer that exhibits desirable properties such as barrier function, biodegradability, oxygen permeability, and good spinnability, enabling the formation of fibres resembling the structure of the-ECM, is polyurethane (PU) [51]. This ECM similarity has been demonstrated to encourage modifications to enhance wound-healing properties. However, the hydrophobic nature of PU limits its adhesion to wounds, thereby reducing its capacity to absorb exudate and protect against infection. The incorporation of natural hydrophilic polymers into polyurethane (PU) matrices has been identified as a solution to the limitations of PU, namely its suboptimal moisture retention and biocompatibility. Rather et al. ([52]) developed fibrous mats loaded with rosemary essential oil (REO) and silver nanoparticles (AgNPs), which were found to enhance wound healing. The incorporation of a carboxylic acid (CA) results in enhanced hydrophilicity and moisture retention, while the presence of PU ensures mechanical stability. The antimicrobial activity was significantly enhanced by the dual action of REO and AgNPs: It has been established that REO is capable of disrupting bacterial membranes and metabolism. In contrast, AgNPs have been shown to generate ROS, release Ag^+^ ions, and damage bacterial DNA and proteins. The combined system demonstrated synergistic antibacterial effects against *S. aureus* and *E. coli*. It is noteworthy that CA reduced the water contact angle from approximately 107° to 26°, thereby facilitating the release and bioavailability of the active agents. This multifunctional composite represents a promising platform for advanced antimicrobial wound dressings [52]. Polyurethane-based nanofibres capitalise on the mechanical properties of the polymer, while natural polymers or drugs contribute biological activity. Furthermore, PU/chitosan nanofibres loaded with linezolid exhibited synergistic properties that promoted effective diabetic wound healing. These nanofibres promoted the infusion and growth of epidermal cells, while improving the distribution and impact of linezolid at the wound site. This dual functionality allows linezolid-loaded nanofibres to accelerate healing and control infections, thus presenting a viable alternative to traditional wound treatment methods [53].

Polyglycolic acid (PGA) is characterised by elevated mechanical strength and is a biocompatible polymer capable of binding water and interacting directly with cells while minimising the risk of immunological reactions [53]. Nevertheless, the accelerated deterioration of PGA can result in localised inflammation, thereby constraining its utilisation. In order to overcome this, a range of modifications are employed [54]. The employment of PGA in the synthesis of nanofibrous membranes, in conjunction with polyurethane and curcumin, has been demonstrated to enhance their regenerative properties, encompassing advantageous mechanical properties, expeditious hygroscopic swelling, and commendable water vapour permeability. In vitro studies have demonstrated that these membranes facilitate high cell viability, while in in vivo models they have been shown to prevent chronic wound formation and supported granulation tissue regeneration [55]. Notwithstanding its low bioactivity and hydrophobicity, which hinder tissue regeneration, poly-ε-caprolactone (PCL) is extensively utilised in wound-healing material synthesis due to its high mechanical strength, biocompatibility, and slow degradation rate [56]. The functionalisation of PCL-based nanostructures with mesoporous materials and curcumin has been demonstrated to enhance their physical properties, improve drug adsorption, support controlled active substance release, and reduce cytotoxicity [57]. The research conducted by Chen et al. (2023) resulted in the development of a gel-nanofibre material that exhibited exceptional mechanical properties, encompassing durability, flexibility, and high air permeability [58]. The slow degradation rate of PCL renders it well suited for use as a scaffold, facilitating the sustained release of active substances. Faraji et al. ([59]) created electro-spun nanofibrous scaffolds made from polycaprolactone (PCL) that were integrated with graphene oxide (GO) and quercetin (Q), with the aim of enhancing wound healing. The incorporation of GO improved the hydrophilicity, porosity, and mechanical integrity of the scaffold, thereby facilitating cellular adhesion, proliferation, and migration. The optimised PCL/GO/Q 0.5 formulation demonstrated a sustained release of quercetin, reaching approximately 70% over a 15-day period. This ensured a continuous delivery of the bioactive compound to the wound microenvironment. The controlled release process has been demonstrated to have a mitigating effect on oxidative stress and inflammation, while concomitantly regulating fibroblast activity and extracellular matrix deposition. Furthermore, the synergistic antibacterial mechanism of quercetin and graphene oxide was attributed to the generation of reactive oxygen species (ROS), which resulted in the disruption of bacterial cell membranes. The multifunctional properties of the subject material have been demonstrated to accelerate wound closure and tissue regeneration. This finding highlights the potential of PCL/GO/Q nanofibrous scaffolds wound-healing applications [59].

Polyethylene glycol (PEG) is synthetic polymer that is frequently employed in chemical modifications. It has been demonstrated to be successful in promoting skin regeneration. The material’s versatility is attributable to three properties: hydrophilicity, biocompatibility and bio-inertness. These properties enable the material to be utilised in a wide variety of combinations. PEG is extensively utilised in drug delivery, cell culture, and tissue regeneration due to its lack of immunogenicity and resistance to protein adsorption [60]. PEG’s potential in wound healing has been demonstrated in studies utilizing chitosan–polyethylene films with copper ions (Cu^2+^). The materials exhibited enhanced mechanical strength and improved wound-healing capacity in comparison to standalone chitosan. This improvement was ascribed to the bonds formed between PEG and chitosan, as well as the antimicrobial properties of copper ions [61]. Similarly, hydrogels based on chitosan crosslinked with polyethylene glycol dicarboxylic acid were biocompatible, self-healing, and facilitated the gradual release of anti-inflammatory agents, enhancing their antibacterial and anti-inflammatory activity [62]. In the context of hydrogel-cellulose nanocomposites, the incorporation of PEG has been demonstrated to enhance the material’s mechanical strength [63].

Synthetic polymers, including PVA, PU, PGA, PCL, and PEG, hold considerable promise in the field of wound therapy. Their ability to form scaffolds, function as drug carriers, and enhance tissue regeneration renders them indispensable in contemporary regenerative medicine. It is recommended that future research focus on the modification and combination of these substances with natural biopolymers. This approach has the potential to enhance their efficiency and expand their applications in wound healing.

## 4. Metal-Based Nanoparticles

Metal-based nanoparticles (MNPs) represent a rapidly developing class of biomaterials with significant potential in the field of wound care. The nanoscale dimensions of the subject are such that there is an enhanced surface-to-volume ratio. This in turn, enables bioavailability to be increased, as well as catalytic activity, and the possibility of surface modification. The properties previously mentioned, when considered in conjunction with the intrinsic biological activities of metals, establish MNPs as multifunctional agents with the capacity to address key challenges in the field of wound healing. These challenges include, but are not limited to infection control, inflammation modulation, and tissue regeneration (see Table 1 for further details).

Among the most extensively studied systems are silver nanoparticles (AgNPs), which exhibit a broad spectrum of antimicrobial activity against Gram-positive, Gram-negative, and multidrug-resistant bacteria [19,64,65]. It is evident that AgNP-based dressings are both safe and reliable, even when used over an extended period of time [66]. Their ability to penetrate biofilms and release silver ions renders them highly promising candidates for the prevention and treatment of infections, thus justifying their incorporation into dressings and combination therapies [20,67].

Gold nanoparticles (AuNPs) have been valued for millennia due to their chemical inertness, biocompatibility, and potential for surface functionalization [68]. The utilisation of these substances in combination therapies, such as photobiomodulation or growth factor delivery, underscores their versatility in enhancing tissue repair processes [69,70,71].In addition to their capacity to support antimicrobial activity, these cells have been shown to regulate angiogenesis, modulate inflammatory cytokines, and serve as carriers for therapeutic molecules [72,73,74,75,76,77]. 

Copper is a trace that plays a vital role in metabolic processes and tissue repair [78]. In nanoparticle form (CuNPs), it has been demonstrated to exhibit antimicrobial, anti-inflammatory, and regenerative activity by enhancing catalytic functions and bioavailability [78,79,80]. CuNPs sized 40–80 nm have been shown to be biocompatible with evidence of promotion of angiogenesis and granulation tissue formation [81]. The antibacterial effect of the compounds under investigation involves membrane interaction, biofilm penetration, and Cu^2+^ ion release, thereby inhibiting resistant strains [82]. In vivo, CuNPs have been demonstrated to accelerate wound healing and reduce inflammation [83].

Zinc-based nanoparticles, with a particular emphasis on ZnO-NPs, with a particular emphasis on due to their antimicrobial, antioxidant, and photoprotective properties [84,85,86,87]. These elements play a pivotal role in metabolic pathways that are indispensable for tissue regeneration and immune response, as well as in the adequate absorption of vitamin A [85,86]. Nevertheless, the size-dependent toxicity of these particles underscores the critical need for nanoparticle engineering to ensure a balance between toxicity and safety [88,89,90,91].

Iron oxide nanoparticles (IONPs) have shown promising applications in chronic wound therapy, particularly in patients suffering from diabetes [92].Their multifunctionality of these substances encompasses antimicrobial, antioxidant, and angiogenesis-promoting properties, along with the ancillary benefit of glucose regulation [93,94]. Importantly, they exhibit broad antimicrobial activity with a low risk of resistance development. However, both iron deficiency and overload have been demonstrated to impair the healing process, thus making careful monitoring of its levels essential [93,95,96].

In summary, MNPs offer multifunctional therapeutic advantages that extend beyond the scope of traditional wound treatments. Whilst the antimicrobial, anti-inflammatory, and regenerative roles of these substances are well documented, their clinical translation is contingent upon the optimisation of physicochemical parameters, functionalisation strategies, and safety profiles.

**Table 1:** Biological properties, mechanisms of action, advantages, limitations, and applications of metal-based nanoparticles in wound healing.

Type of Nanoparticles	Biological Properties	Mechanisms of Action	Advantages in Wound Therapy	Limitations/Toxicity	Examples of Applications
Silver (AgNPs)	Antibacterial, anti-inflammatory, promoting keratinocyte proliferation and fibroblast differentiation [64]	(a) Increasing bacterial membrane permeability and protein loss (b) ROS generation (c) DNA replication disruption [21,64,67]	Active against Gram+ and Gram– bacteria, including resistant strains; long-lasting efficacy; synergy with collagen [20]	Systemic Ag^+^ accumulation in vital organs poses toxicity risks; cytotoxicity risk at high doses (>10 µg/mL) [21]	AgNP-based dressings, penetration of *E. coli* biofilms (20 nm) [67]
Gold (AuNPs)	Biocompatible, anti-inflammatory, angiogenesis-modulating, antibacterial [72]	Surface functionalization (ligands, drugs, growth factors); cytokine regulation (IL-6, TNF-α) [69,70]	Multifunctionality: stimulation of angiogenesis, proliferation, stem cell differentiation; improved mechanical stability of composites [75,76,77]	Debated toxicity; some gold compounds harmful (AuCl_3_, KAu(CN)_2_) [71]	Collagen composites, AuNPs + KGF coatings, APA-AuNPs wound dressings active in vivo [73,74]
Copper (CuNPs)	Antibacterial, anti-inflammatory, pro-angiogenic, regenerative [78]	Cu^2+^ release damages bacterial membranes; cofactor for enzymes (SOD, cytochrome oxidase); VEGF induction [79,80]	Supports fibroblast proliferation, endothelial cell migration; accelerates granulation tissue formation [81]	Excess copper → oxidative stress, lipid peroxidation, cytotoxicity [78]	CuNP-based dressings (40–80 nm, non-toxic), active against biofilms [81,82]
Zinc (ZnO-NPs)	Antibacterial, antioxidant, supports keratinocyte proliferation and metabolic processes [85]	Alkaline phosphatase activity → adenosine formation (anti-inflammatory); ROS; UV blocking [85]	Wide dermatological use; accelerates wound contraction; antimicrobial and photoprotective [84,87]	Small ZnO-NPs (<50 nm) may be cytotoxic (apoptosis, ROS, genotoxicity); larger (>100 nm) are biocompatible [88,89]	Modified ZnO-NPs in wound dressings, beneficial in infected wounds [90,91]
Iron (IONPs)	Antibacterial, antioxidant, antifungal, promotes proliferation and angiogenesis [94,96]	Involvement in metabolism (heme, DNA, cell cycle); ROS regulation; α-amylase inhibition (benefits in diabetes) [92,93]	Accelerates diabetic wound healing; supports angiogenesis; difficult for bacteria to develop resistance [94,95]	Excess iron → oxidative stress, tissue damage; deficiency → anemia, slower healing [92,93]	Hydrogels with IONPs (cellulose + gelatin), antibacterial activity against MDR strains [94]

## 5. Lipid Nanoparticles

The utilisation of lipid nanoparticles as carriers for a variety of therapeutic agents, including drugs, growth factors, and small interfering RNA (siRNA) has been demonstrated to be a promising area of research [97,98]. The initial lipid nanoparticles, designated as solid lipid nanoparticles (SLNs), proffer numerous benefits, encompassing physical stability, protection of drugs from degradation, controlled release, and minimal cytotoxicity, contingent upon the utilisation of well-tolerated excipients are used [99]. However, SLNs are not without their limitations, such as low drug-loading capacity and premature drug expulsion during storage [100]. To overcome these limitations, second-generation lipid nanoparticles, known as nanostructured lipid carriers (NLCs), were developed. It has been demonstrated that NLCs are biocompatible, and that they protect active agents from degradation. Furthermore, it has been established that NLCs release drugs in a controlled manner, and that they possess moisturising properties for the skin. Moreover, NLCs have been shown to exhibit a higher degree of drug entrapment efficiency in comparison to SLNs. Indeed, NLCs have been shown to achieve up to 88% drug entrapment in contrast to the approximately 80% achieved by SLNs. These properties render NLCs particularly promising candidate for wound healing and skin regeneration. It is evident that both SLNs and NLCs offer numerous advantages over traditional carriers. Furthermore, their small size allows them to penetrate biofilms, thereby enhancing the efficacy of drugs against bacterial cells [101].

For instance, the antimicrobial peptide LL37, which plays a critical role in defence against infections, was delivered to wounds via SLNs. The release of the peptide occurred in a gradual manner over a period of 14 days. It was observed that the stability of the peptide was significantly enhanced and its degradation was effectively prevented by the SLNs. The continuous LL37 release exhibited potent bactericidal effects against *S. aureus* and *E. coli*, accelerating wound healing. The antibacterial activity of LL37-loaded SLNs is attributable to the peptide’s electrostatic interaction with negatively charged bacterial membranes, resulting in membrane disruption. The lipid matrix has been demonstrated to play a pivotal role in the protection of LL37 from degradation, ensuring its sustained release and enhancing biofilm penetration. This, in turn, has been shown to result in a reduction in microbial load, attenuation of inflammation, and acceleration of re-epithelialization in vivo [102]. In a similar manner, cumin oil-loaded NLCs exert their therapeutic effects through the gradual release of active phytochemicals, such as carvone and limonene. These phytochemicals have been shown to destabilise bacterial membranes and inhibit metabolic enzyme activity. The topical application of this therapeutic material was found to reduce inflammation by suppressing TNF-α and IL-1β expression, promote the proliferative phase by decreasing MMP-3 expression, and facilitate granulation tissue formation [103].

Both SLNs and NLCs undergo modifications to enhance drug delivery and release at the wound site, thereby promoting skin regeneration [104]. The accelerated wound healing process is attributed to the local release of active compounds from SLNs loaded with chamomile. This results in a reduction in IL-1β levels, a key cytokine in the inflammatory process, and an increase in TGF-β1 levels during the proliferative phase. These effects ultimately lead to wound closure [105]. NLCs also serve as lipid components in advanced wound dressings. In a dressing composed of aloe vera and poly(lactic-co-glycolic acid), NLCs prevented the adhesion of the dressing to the wound, thus facilitating its removal after tissue regeneration. This approach has been demonstrated to enhance tensile strength and exudate absorption, thereby accelerating wound healing and improving dressing adhesion [106].

In summary, lipid nanoparticles, specifically SLNs and NLCs, demonstrate considerable promise in the field of wound therapy due to their biocompatibility, controlled drug release capabilities, and ability to penetrate bacterial biofilms. These materials have been shown to be more successful at promoting tissue regeneration and infection control when they are modified and integrated into wound dressings.

## 6. Polyphenols

Polyphenols are synthesised exclusively by plants and serve as secondary metabolites. These natural compounds are present in all plant species and are responsible for a variety of vital functions. The function of these structures and organisms is threefold: firstly, to attract pollinators; secondly, to provide structural support; and thirdly, to protect against ultraviolet radiation and safeguard plants from microbial invasions and herbivores [107]. In the context of food products, polyphenols have been shown to contribute to the sensory and nutritional properties of plant-based foods, with effects including astringency, colour, and aroma, depending on their concentration [108]. Additionally, it has been demonstrated that certain polyphenols have the capacity to bind to macromolecules, including but not limited to carbohydrates and digestive enzymes, which may result in a reduction in food digestibility [109]. A significant focus of research has been directed towards investigating the antioxidant, antimicrobial, antiviral, antibacterial, anti-inflammatory, anticancer properties of these substances. Furthermore, considerable attention has been given to their capacity to modulate specific signalling pathways within the human body. Polyphenols have been shown to protect cells from oxidative stress by neutralising reactive oxygen species (ROS) and activating antioxidant enzymes such as superoxide dismutase (SOD), catalase, and glutathione peroxidase. Furthermore, the anti-inflammatory effects of these compounds are attributed to their capacity to inhibit the NF-κB and MAPK signaling pathways, thereby reducing the expression of pro-inflammatory cytokines, including TNF-α and IL-6. The antimicrobial and antiviral activities of these substances are a result of the disruption of pathogen cell membranes, interference with metabolic processes, and inhibition of viral replication. In addition, polyphenols have been demonstrated to possess anticancer properties, which are characterised by the induction of apoptosis, the suppression of cancer cell proliferation, and the modulation of the PI3K/Akt and p53 signalling pathways. It has been established that, collectively, these mechanisms regulate key cellular mechanisms, thus maintaining homeostasis and protecting the organism against degenerative diseases [107]. Table 2 summarizes the key physicochemical properties, bioavailability profiles, therapeutic effects, and limitations of selected polyphenols used in wound healing strategies.

Polyphenols have been shown to possess high antioxidant activity, which protects against ROS by neutralising free radicals. In addition, polyphenols have been demonstrated to have antimicrobial potential against bacteria found in infected chronic wounds [110]. Although the precise mechanisms through which polyphenols exert their antimicrobial activity remain to be fully elucidated, it has been hypothesized that they may cause bacterial cell wall disruption through the hydrophobic components of phenolic compounds, interfere with intracellular functions by forming hydrogen bonds with enzymes, or modify cell wall structures, leading to loss of integrity due to interactions with the cell membrane [111]. The lipophilic character of polyphenols is known to be enhanced, which has been shown to increase their antibacterial activity. This is achieved by promoting membrane penetration, leading to disruption of lipid bilayer integrity and inhibition of membrane-associated enzymatic functions. QSAR analyses indicate that polyphenols with moderate lipophilicity (optimal log *p* ≈ 2–4) exhibit the highest antibacterial potential, achieving an optimal balance between membrane affinity and aqueous solubility [112]. Studies suggest that these bioactive plant compounds may combat antibiotic-resistant bacterial strains, such as methicillin-resistant *S. aureus* [113]. In addition to their antioxidant and antimicrobial properties, polyphenols also exhibit metal-chelating abilities. The presence of an aromatic ring and specific functional groups, namely carboxyl, carbonyl, and hydroxyl has been demonstrated to facilitate the binding o metal ions by polyphenols [114]. Chelating transition metals, such as iron or copper have been shown to reduce the rate of Fenton reactions. This effect is believed to be a means of preventing oxidation induced by reactive hydroxyl radicals. This allows polyphenols to protect cells from oxidative damage, support detoxification processes, and improve the biological stability of organisms [115].

**Figure 4:**
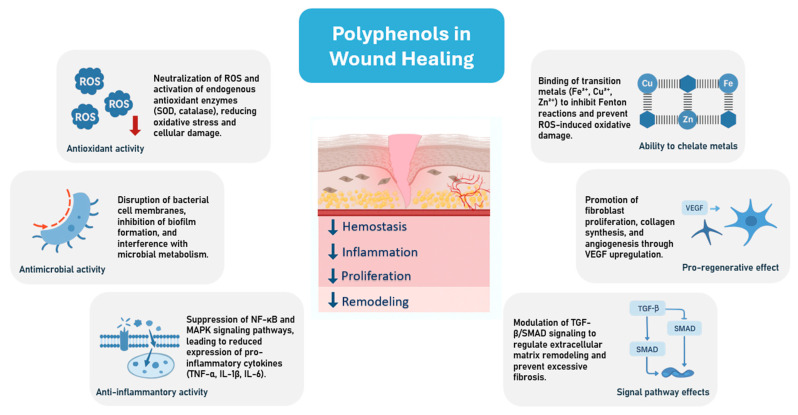
Mechanisms of polyphenol action in wound healing.

The favourable properties of polyphenols have prompted researchers to incorporate them into dressings for the treatment of chronic wounds (Figure 4). For example, thymol, due to its antioxidant and antimicrobial properties, has been incorporated into materials such as films and hydrogels. A combination of bacterial cellulose and thymol was demonstrated to produce a robust antimicrobial response against pathogens specific to burns. It has been established that thymol is capable of disrupting bacterial cell membranes. This disruption occurs as a result of the substance integrating into the lipid bilayer, thereby increasing membrane permeability. Its antioxidant capacity allows for the neutralisation of ROS and the downregulation of inflammatory cytokines such as TNF-α and IL-1β, thereby mitigating oxidative stress and inflammation. It has been demonstrated that the porous and hydrophilic bacterial cellulose network has the capacity to support fibroblast adhesion, proliferation, and collagen synthesis, whilst maintaining a favourable moisture balance at the wound site. In vivo, the combined effects result in enhanced re-epithelialisation, improved angiogenesis and increased wound contraction when compared to untreated controls [116].

Consequently, the beneficial properties of tannic acid have prompted researchers to develop a hydrogel containing free tannic acid and trimethylolpropane triglycidyl ether as a crosslinking agent, which has been designed for the treatment of chronic wounds. The hydrogel exhibited a satisfactory antioxidant and antimicrobial profile. It exhibited strong antibacterial activity against Gram-positive *Staphylococcus aureus* and *Bacillus subtilis*, Gram-negative *Pseudomonas aeruginosa*, and the fungal strain *Candida albicans*. Cytotoxicity studies conducted on L929 fibroblast cell lines in vitro suggested that the hydrogel is non-toxic [117].

In contrast, quercetin has been shown to possess the capacity to attenuate fibrosis and scarring during the wound healing. The study demonstrated that the substance promoted fibroblast cell proliferation, decreased immune cell infiltration, and triggered changes in signalling pathways associated with fibrosis [118].

In one study, a sponge composed of chitin nanofibres was developed, with a tannic acid–calcium (Ca^2+^) complex anchored to its surface. The porous nature of the sponge, in conjunction with the presence of a complex, has been demonstrated to enhance platelet activity, thereby reducing hemostasis time in both in vitro and in vivo models. Additionally, the combination of tannic acid and calcium improved the antibacterial properties of the sponge, with its activity depending on the concentration of the complex present. The antibacterial activity of the TA/Ca^2+^ sponge is attributable to three main factors. Firstly, bacterial membrane disruption is a key element in the mechanism of action. Secondly, metal ion chelation is essential for bacterial metabolism. Thirdly, enhanced electrostatic interactions between TA/Ca^2+^ complexes and bacterial cell surfaces are also a contributing factor. It has been demonstrated that these mechanisms act in a synergistic manner, resulting in the substantial elimination of both Gram-positive and Gram-negative bacteria. [119]. Tannic acid has also been utilized in various combinations, including as a component of biocompatible nanofibres (NFs) containing chitosan and pullulan. These nanofibers have been demonstrated to exert synergistic effects against the Gram-negative bacterium *Escherichia coli*. The tri-component composite was crosslinked to ensure water stability, making it a potential wound dressing. The composite membrane exhibited a high water-absorption capacity with rapid uptake and supported fibroblast adhesion and growth. The creation of a three-dimensional environment mimicking the ECM in the skin was achieved, thereby enabling cell migration through the fibrous structure. This phenomenon has been shown to promote interlayer growth throughout the membrane, thereby facilitating deeper and more complex wound healing [120].

Another notable polyphenol, hydrophobic curcumin, is characterised by its antimicrobial, antioxidant, and wound-healing properties. However, its limited water solubility and rapid rate of degradation limit its potential application. To overcome these barriers, curcumin was encapsulated in a sialic hydrogel carrier. These nanoparticles have been demonstrated to significantly inhibit the growth of methicillin-resistant *S. aureus* and *P. aeruginosa* growth in vitro, while concomitantly improving the healing of full-thickness burn wounds in a mouse model. Treatment with nanocurcumin has been demonstrated to result in enhanced wound contraction, accelerated re-epithelialisation, and increased collagen deposition when compared to both free curcumin and control groups. The formulation the ability to reduce levels of inflammatory cytokines (TNF-α, IL-1β and IL-6) by inhibiting NF-κB signalling. In addition to this, the formulation also stimulated angiogenesis and fibroblast proliferation [121]. In addition, a combination of curcumin and a silver nanohydrogel with aloe vera was employed. This composite was designed to exhibit antimicrobial properties, support wound healing, and control infections. The performance of the composite was evaluated based on its ability to inhibit the growth of *E. coli* and *S. aureus* growth. The experimental results demonstrated that the incorporation of silver nanoparticle-based dressings resulted in a significant reduction in bacterial proliferation, with a maximum decrease of up to 98% observed in both cases when compared to the control group. These findings suggest the potential of nano-silver-containing dressings for controlling infections at wound sites. In vivo studies on a mouse model showed that after 16 days, composite dressing produced a greater reduction in wound size than the control [122].

Despite their promising properties, polyphenols face limitations such as instability, low water solubility, poor bioavailability, light sensitivity, and limited cellular membrane permeability [123]. Under conditions of low light exposure, polyphenols are susceptible to oxidation, a process which complicates their utilisation, management, and delivery to target tissues. Additionally, the concentrations of phenolic compounds that have been demonstrated to be effective in in vitro tests have frequently been found to be insufficient in in vivo studies [124]. To address these issues, encapsulating polyphenols as a delivery system can improve their stability and control their release and targeted delivery [125]. Studies comparing the activity of encapsulated versus free-form polyphenols have demonstrated superior effects for the former [126]. For example, the use of curcumin encapsulated in a hydrogel to promote wound healing in diabetic patients has been shown to be significantly more successful [127]. Similarly, although resveratrol offers potential health benefits, its use in commercial pharmaceutical products is hindered by poor water solubility, low bioavailability, and chemical instability. In this context, nanocarrier systems utilising pectin have been investigated for their ability to protect and deliver phenolic compounds. The antioxidant activity of resveratrol was found to be enhanced by encapsulation in comparison with its free form [128].

Despite their therapeutic potential, polyphenols face challenges such as low water solubility, poor bioavailability, and instability. These limitations can be addressed through encapsulation technologies, particularly using chitosan-based carriers, which allow for controlled release, protection of active compounds, and targeted delivery to the wound site.

The incorporation of polyphenols into biomaterials, including hydrogels, sponges, and nanofibers has been demonstrated to facilitate tissue regeneration, enhance therapeutic outcomes, and reduce the risk of infection, making them promising components in advanced wound dressings.

**Table 2:** Key physicochemical and therapeutic characteristics of selected polyphenols relevant to wound healing applications.

Polyphenol	Solubility	Bioavailability	Main Biological Effects	Limitations
Curcumin	Very low in water [120]	Low [122]	Antioxidant, antimicrobial, anti-inflammatory, supports tissue regeneration [120,121]	Poor solubility, chemical instability, low absorption [120,122,127]
Quercetin	Low [117]	Low–moderate (improved when encapsulated) [126]	Antioxidant, reduces fibrosis, promotes fibroblast proliferation [117,126]	Low solubility, photo-instability, limited permeability [122]
Tannic acid	Mederaye (hydrophilic) [116,119]	Moderate [116]	Antimicrobial, antioxidant, hemostatic, enhances fibroblast activity [116,118,119]	Astringency, potential for protein binding [108]
Resveratrol	Poor [128]	Very low [128]	Antioxidant, anti-inflammatory, promotes angiogenesis [128]	Unstable, low water solubility, rapid degradation [128]
EGCG	High [109]	Moderate to high [109]	Antioxidant, antibacterial (incl. against MRSA), anti-inflammatory [109]	Light-sensitive, requires stabilization [122]

## 7. Integrated Approach to Wound Healing—Modification of Chitosan Nanocomposites

In light of the multifactorial nature of chronic wounds, the development of an integrated strategy that incorporates the most effective biomaterials has emerged as a promising direction. In order to synthesize the findings previously discussed, Section 7 provides a comparative overview of seminal studies on modified chitosan nanocomposites. This section emphasizes the synergistic mechanisms between polyphenols and nanostructured carriers.

A novel, integrated approach to wound treatment that combines chitosan nanocomposites with polyphenols such as quercetin, epigallocatechin gallate, and curcumin, metals or other nanostructures offers improvements and prospective approaches in wound therapy. The synergy of these components has been demonstrated to enhance healing, reduce infection, and improve overall tissue regeneration [129]. As demonstrated in Table 3, a comparative analysis reveals that the integration of polyphenols, such as quercetin or EGCG, into chitosan nanocomposites leads to a more pronounced enhancement of angiogenesis and a more significant reduction of inflammation in comparison to unmodified systems. This observation suggests the presence of a synergistic mode of action.

**Table 3:** Summary of original studies on chitosan-based composite biomaterials and nanoparticles in wound healing: composition, cellular models, and biological outcomes.

Material	Experimental Model	Key Mechanisms	Outcomes	References
Chitosan–AgNPs with calendula extract	Clinical trials (patient with chronic wounds)	Antibacterial, anti-inflammatory, pro-regenerative	Reduced infection rate and inflammation, accelerated wound closure (fully healed after 4 months)	[28]
Chitosan–Zn complex films	In vitro (ST-2, RAW 264.7, HaCaT, MEF cells)	Antibacterial, angiogenic, anti-inflammatory	Antibacterial activity against *S. aureus* (near-complete elimination at 24 h) increased (~30%) VEGF secretion, moderately decreased NO release, low cytotoxicity, improved wound closure (88% closure)	[130]
Chitosan-based matrices containing silver nanoparticles (AgNPs@Chi)	In vitro (HaCaT, NIH/3T3 cells), in vivo (mouse model)	Sustained Ag^+^ release, antifungal activity, biocompatible, reduced hemolysis	Continuous Ag^+^ release over 52 days, reduced infection, enhanced fibroblast and keratinocyte proliferation, accelerated regeneration, inhibition of fungal growth (90%)	[131]
Chitosan–PVA–Ag nanoparticles	In vitro (CHO-K1 cells), in vivo (rat model)	Antibacterial, antioxidant	Low cytotoxicity (5–200 μg/mL), promoted re-epithelialization, accelerated early-stage wound healing (100% of wound closure after 12 days)	[132]
Chitosan–graphene hydrogel	In vivo (rat model)	Self-healing, hemostasis, adhesive	Accelerated re-epithelialization, complete wound closure in 10 days, improved mechanical properties and biocompatibility	[133]
Chitosan–collagen sponge	In vitro (NIH3T3 cells), in vivo (rat model)	ECM support, enhanced fibroblast proliferation, improved cell adhesion, antibacterial	Enhanced epithelialization (95%) and antibacterial activity against *E. coli* i *S. aureus*, high water retention, low cytotoxicity	[134]
Chitosan–alginate–ZnO	In vitro (3T3, 293T cells), in vivo (rat model)	Broad-spectrum antibacterial, biocompatibility	Biocompatible, strong antibacterial activity against Gram-positive and Gram-negative bacteria, antifungal activity against *C. albicans*, sustained Zn^2+^ release, accelerated wound healing	[135]
Chitosan–polyvinylpyrrolidone–dihydroquercetin	In vitro (HaCaT cells), in vivo (mouse model)	Antioxidant, antibacterial, pro-angiogenic, supported re-epithelialization	Fast wound closure, ↑ VEGF, CD31, pan-keratin expression, enhanced tissue regeneration	[136]
Chitosan–hyaluronate–resveratrol sponge	In vitro (HFL cells), in vivo (mouse model)	Biocompatible (~80% viability), pro-regenerative, bacteriostatic, accelerated re-epithelialization	Enhanced granulation tissue formation and vascularization, promoting angiogenesis, accelerated wound closure (>50% wound area reduction by day 10 vs. control)	[137]
Chitosan–gelatin nanoparticles with EGCG and ascorbic acid	Diabetic mice (ICR)	Anti-inflammatory, angiogenic, accelerated re-epithelialization	Promoted collagen accumulation, angiogenesis, ↓ inflammation (reduced macrophage infiltration), improved wound closure	[138]
Chitosan–quercetin nanoparticles	In vivo (rat model)	Angiogenic, modulation of the inflammatory phase, accelerated fibroblast activity and ECM remodelling.	↓ TNF-α, ↑ IL-10, VEGF, and TGF-β1 expression, improved granulation and collagen deposition	[139]
Chitosan–clotrimazole–grape extract nanoparticles	In vivo (rat model)	Antifungal, antioxidant, hemocompatibility, synergistic bioactivity	Sustained release, improved bioavailability, complete tissue repair after 7 days, antifungal activity—inhibition zones of 72 mm (*A. niger*) and 74 mm (*C. albicans*), high entrapment efficiency (94.7%)	[140]
Tripolyphosphate–chitosan–curcumin nanoparticles	In vitro (HDF cells), in vivo (rat model)	Antibacterial, antioxidant, pro-regenerative, re-epithelialization	Enhanced cell proliferation, reduced infection (99% inhibition), improved tissue organization and wound healing (96–99% wound closure)	[141]

**Figure 5:**
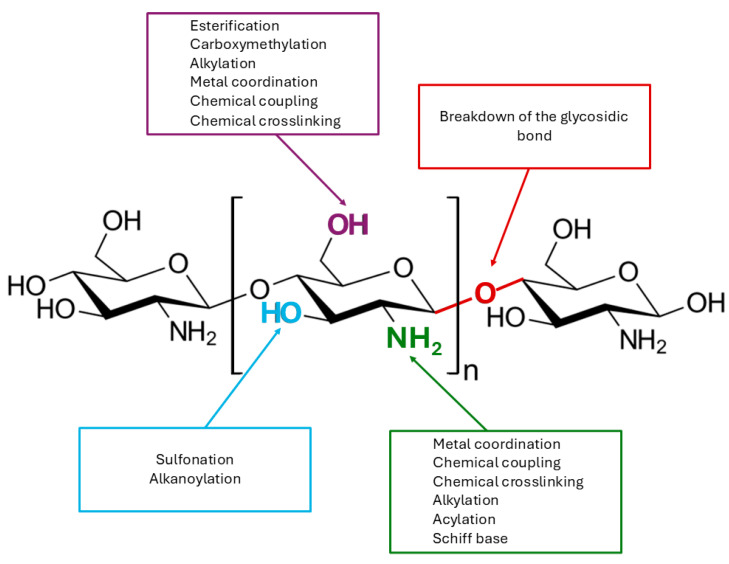
Chemically modifiable functional groups present in the structure of chitosan. Reprinted from [143], an open access article distributed under the terms of the Creative Commons CC BY license (http://creativecommons.org/licenses/by/4.0/ (accessed on 29 October 2025)) (modified).

The structural characteristics of chitosan, which include the presence of reactive functional groups such as amino and hydroxyl groups, enable the material to undergo a wide range of modifications. These modifications can be categorised into several different types, including metal coordination, chemical conjugation, cross-linking, graft copolymerisation, and alkylation and among others. The glycosidic linkages and the acetamide group present in the polymer can also be considered as functional groups (Figure 5) [142]. The presence of these functional groups enables a multitude of modifications, thus facilitating the development of polymers with novel properties and characteristics.

Modifications to the chemical structure of chitosan, for example, by the introduction of metal ions, have been demonstrated to enhance its capacity to promote wound healing and augment its antibacterial properties. Chitosan–zinc complexes in the form of homogeneous, smooth films exhibit strong, quantitatively confirmed antibacterial activity against *S. aureus*, including the near-complete elimination of colonies after 24 h. However, they demonstrate weaker effects against *E. coli*. These materials, particularly 12ChiZn, demonstrate measurable pro-angiogenic responses, such as increased VEGF secretion and moderate nitric oxide reduction, indicating anti-inflammatory potential. Furthermore, the complexes—most notably 6ChiZn—significantly accelerate keratinocyte migration and in vitro wound closure in HaCaT scratch assays. Cytocompatibility analyses revealed no toxicity in indirect tests (ST-2 and RAW 264.7). 6ChiZn was found to provide an optimal balance of cellular viability and bioactivity, whereas 12ChiZn displayed some degree of cytotoxicity when in direct contact with fibroblasts [130].

Chitosan modifications also include its combination with gold, which exhibits desirable biocompatibility, stability, and a high surface-to-volume ratio, making it ideal for applications in medicine and tissue engineering. It has been demonstrated that gold exhibits stability within biological tissues and functions to impede the biodegradation of nanoparticles [144]. The porous 3D network structure of hydrogels has been demonstrated to facilitate wound healing by means of exudate absorption and the provision of a moist, breathable environment. Furthermore, these structures have been demonstrated to accelerate the healing of chronic wounds by means of delivering bioactive molecules [145].

The antibacterial effects of AgNPs are limited by their high toxicity towards human cells at concentrations sufficient to eliminate microorganisms [1]. In order to combat bacteria while reducing toxicity, biocompatible polymers such as chitosan are used as matrices for AgNPs. Chitosan, with its inherent antibacterial properties, biodegradability, and non-toxicity, plays a dual role in this regard: it enhances the antimicrobial activity of AgNPs and provides biocompatibility for the entire material. Chitosan’s structural characteristics enable the retention of silver nanoparticles while allowing their controlled release, thereby enhancing antimicrobial performance and reducing toxicity to human cells [1,3,146]. Chitosan-based matrices containing silver nanoparticles (AgNPs@Chi) have attracted attention due to their ability to reduce silver’s toxicity while maintaining its antimicrobial activity. Chitosan enabled the sustained and complete release of Ag^+^ over 52 days, ensuring prolonged antimicrobial activity. The functionalisation of AgNPs with chitosan significantly reduced hemolysis, lowering it to 15–18% at concentrations below 1 µg/mL, compared to 20–30% for unmodified nanoparticles. AgNPs@Chi also enhanced fibroblast and keratinocyte proliferation, achieving complete scratch closure within 48 h at a concentration of 1 µg/mL of Ag in HaCaT assays. AgNPs exhibited strong inhibitory effects on fungal growth, achieving ≥ 90% inhibition at a concentration of 0.12 µg/mL. In contrast, chitosan-functionalised AgNPs (AgNPs@Chi) only produced a comparable effect at higher concentrations (0.5–1 µg/mL). In mouse models, AgNPs@Chi markedly reduced infection and accelerated tissue regeneration. Therefore, the combination of silver with chitosan enhances antimicrobial and regenerative performance while reducing toxicity, resulting in a superior therapeutic impact [131]. The combination of AgNPs with chitin derivatives has been shown to promote wound healing by combining antibacterial and antioxidant activities in a synergistic way. Chitosan–PVA–silver nanoparticles (CS-AgNPs) exhibited markedly enhanced antioxidant capacity, an improvement that can be attributed to the functionalisation of the chitosan with silver nanoparticles, which significantly reinforced its radical-scavenging efficiency. Furthermore, CS-AgNPs demonstrated robust broad-spectrum antibacterial activity against both Gram-positive and Gram-negative bacteria. The inhibition zones increased proportionally to the silver content, surpassing the performance of chitosan alone. The nanocomposite also showed clear safety advantages, including minimal haemolysis (≤1% at 1–3 mg/mL) and low cytotoxicity towards CHO-K1 cells. Only modest growth inhibition was observed at 5–20 μg/mL, with approximately 26% inhibition at 200 μg/mL. This indicates improved biocompatibility relative to pure AgNPs. CS-AgNPs were also found to significantly accelerate wound healing, achieving 73–79% contraction by day 9 and complete closure (97–100%) by day 12, substantially outperforming both untreated wounds and those treated with conventional formulations. Histological analysis further confirmed superior regeneration characterised by full re-epithelialisation, well-organised granulation tissue, dense neovascularisation and the absence of inflammatory infiltrates. Taken together, these findings suggest that CS-AgNPs offer several enhancements, including stronger antioxidant and antibacterial effects, reduced toxicity, faster wound contraction and more thorough tissue restoration [132].

Researchers continue to modify chitosan with materials that are already used in medicine. For instance, combining hydrogels with graphene has been shown to provide excellent adhesion and hemocompatibility. Incorporating graphene enhances the hydrogel’s internal pore structure, absorption capacity and mechanical strength, while the dynamic and reversible breaking and recombination of non-covalent bonds between chitosan and graphene oxide gives it self-healing and injectable properties. Further adjustment of the graphene content allows precise control over the hydrogel’s mechanical and rheological behaviour. In vivo studies using a full-thickness wound model in rats revealed that the material achieved almost complete wound closure within 10 days, outperforming both untreated controls and commercially available Tegaderm™ dressings. Furthermore, the hydrogel significantly reduced blood loss and hemostatic time, lowered inflammatory cell infiltration, promoted re-epithelialisation and facilitated dense, well-organised collagen deposition, indicating superior tissue regeneration [133].

Zhang et al. ([134]) developed a novel wound-healing and tissue-regeneration dressing in the form of a collagen-enriched chitosan sponge (CCS). The prepared CCS dressing demonstrated low toxicity towards NIH3T3 cells and exhibited significant antibacterial activity against both *E. coli* and *S. aureus*, in comparison with the control (non-collagen chitosan sponge). This composite material, formed by blending chitosan with type I collagen, displayed several advantageous characteristics. Firstly, it demonstrated an excellent capacity to retain water, maintaining a moist environment that is essential for accelerated tissue repair. Secondly, the dressing showed high biocompatibility and strong moisture-retention properties due to the hydrophilic nature of chitosan. Thirdly, in vivo experiments revealed that CCS markedly accelerated re-epithelialisation: the wound area in the CCS-treated group was significantly smaller on days 3, 7 and 14 than wounds treated with chitosan or collagen alone. Furthermore, CCS achieved 95% re-epithelialisation in a significantly shorter timeframe (13.8 ± 0.75 days) than either chitosan (17.0 ± 0.82 days) or collagen (15.3 ± 0.50 days). Histological evaluation further demonstrated that CCS promoted more orderly fibroblast proliferation, increased neovascularisation, reduced inflammatory cell infiltration and facilitated denser, more organised collagen deposition. Overall, these results suggest that the CCS dressing promotes superior wound closure and enhanced tissue regeneration compared to the individual components used separately [134].

The combination of chitosan with another marine-derived carbohydrate, alginate, has been shown to enhance the valuable properties of both substances, including biocompatibility, low immunogenicity, water retention, and biodegradability, thereby creating materials with enhanced functionality [147]. However, the low mechanical and antibacterial properties of alginate hydrogels restrict their potential applications. This hydrogel exhibited broad-spectrum antibacterial activity against both Gram-positive and Gram-negative bacteria, as well as fungi. This included *E. coli*, *C. albicans*, *S. aureus* and *B. subtilis*. Notably, it demonstrated particularly strong inhibition against B. subtilis, achieving an inhibition zone of 3.1 cm. The composite was also biocompatible with blood cells and the 3T3 and 293T cell lines. It showed low hemolysis (1.3–2.4%) and maintained over 80% cell viability. The sustained release of Zn^2+^ ions contributes to the material’s long-lasting antimicrobial action. In vivo studies have also demonstrated that it significantly accelerates wound healing, resulting in faster re-epithelialisation, reduced inflammation, enhanced collagen deposition and visible regeneration of skin appendages [135].

Chitosan nanofibres closely resemble the natural extracellular matrix; however, the high viscosity of chitosan solutions complicates the production of pure chitosan fibres, and scaffolds made solely from chitosan exhibit inadequate mechanical properties and low structural stability in aqueous environments [148]. To address these challenges, the combination of chitosan with other biocompatible polymers is often employed. Curcumin and piperine were successfully encapsulated in zein–chitosan nanoparticles to form stable, spherical particles measuring approximately 500 nm. These particles exhibited high encapsulation rates of 89% and 87% for curcumin and piperine, respectively. The formulation exhibited significant cytotoxicity against neuroblastoma cells, with concentrations of 10–25 µg/mL reducing cell viability by approximately 50%. Encapsulation enhanced the stability and therapeutic potential of curcumin, establishing this natural nanoparticle system as a promising platform for improved bioactive delivery [149].

In the treatment of chronic diabetic wounds, carboxymethyl chitosan modified with four-arm PEG benzaldehyde and a solution containing basic fibroblast growth factor (bFGF) has shown potential for addressing delayed wound repair. Conventional clinical strategies, including tissue debridement, skin grafting, blood glucose control, and pressure reduction—often prove ineffective in the absence of wound-healing process activation in diabetic patients. The combination of these advanced materials and techniques has the potential to overcome the limitations of current treatments and provide innovative solutions for wound management. Chitosan hydrogel dressings exhibit strong adhesion to moist tissues, self-healing properties, and antibacterial effects. The findings of biological analysis demonstrate that these composites exhibit a high degree of biocompatibility, rapidly arresting bleeding and significantly accelerating the healing process of full-thickness diabetic wounds. The process is realised through an augmentation in the production of Ki67, a protein that is present in cell nuclei during periods of active proliferation. This process supports new epithelium and collagen formation, induces hair follicle development, and enhances neovascularization through elevated production of CD31 and CD34 markers. These characteristics underscore the composite’s promise in the management of chronic diabetic wounds. The capacity to modify chitosan with materials such as metals or polyphenols facilitates the fabrication of dressings with specialised properties [150].

**Figure 6:**
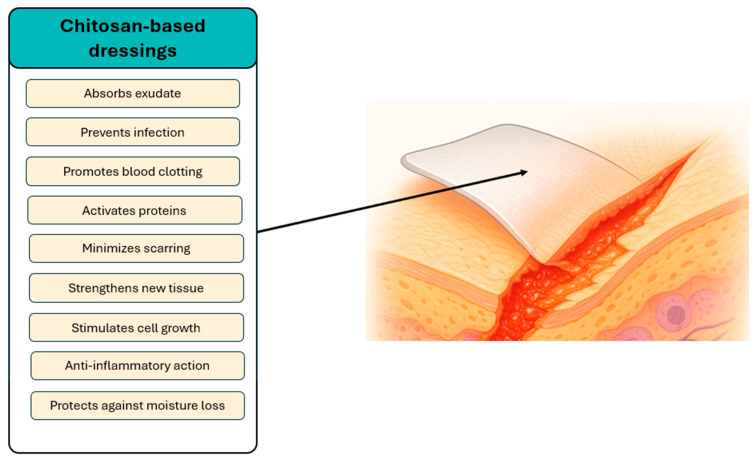
Key properties of chitosan dressings modified with polyphenols.

The utilisation of combinations of polyphenols and chitosan is frequently linked to the occurrence of synergistic effects that facilitate wound healing. A film composed of chitosan, PVP (polyvinylpyrrolidone) and dihydroquercetin (DHQ) has been demonstrated to possess favourable morphological properties, thermal stability and hydrophilicity. These properties render the film suitable for utilisation in wound treatment applications. The film demonstrated its capacity to combat *S. aureus* and *E. coli* bacteria, as well as its ability to scavenge DPPH free radicals, thereby substantiating its antioxidant activity. The MTT assay was employed to assess the toxicity of the film on HaCaT cells. The results of this assay demonstrated that the film is non-toxic to HaCaT cells, thereby confirming its safety. In vivo experiments demonstrated that wounds treated with the CS-PVP-DHQ nanofibre film exhibited accelerated healing, thereby underscoring its potential to expedite tissue regeneration. Furthermore, the nanofiber film induced autophagy pathways and increased the expression of pan-keratin, VEGF, and CD31, suggesting that it supports biological processes related to tissue regeneration and wound healing [136]. Modifications of chitosan can enhance its existing properties or introduce entirely new functionalities (Figure 6).

It is well established that polyphenols frequently demonstrate inadequate water solubility, a property which invariably curtails their bioavailability. The encapsulation of the compound within chitosan nanoparticles (CS-NPs) has been demonstrated to enhance their solubility, a property that is essential for optimal absorption within the body. The utilisation of CS-NPs facilitates the regulated release of polyphenols, thereby ensuring the sustained delivery of active compounds. The consequence of this is a reduction in the frequency of drug administration and a minimisation of side effects. Furthermore, CS-NP modification to target specific cells or tissues has been shown to increase the success of treatment while reducing damage to healthy tissue. CS-NPs are a pioneering development in the fields of medicine and pharmacology, with the potential to enhance the outcomes of polyphenol-based therapies [151].

Berce et al. ([137]) synthesised a polymer containing chitosan, sodium hyaluronate, and resveratrol with a view to evaluating its regenerative potential. The sponge was then subjected to a series of tests to ascertain its toxicity levels against a human lung fibroblast cell line (HFL). Berce et al. ([137]) synthesised a polymer containing chitosan, sodium hyaluronate and resveratrol, with the aim of evaluating its regenerative potential. The sponge was then subjected to a series of tests to determine its toxicity against a human lung fibroblast cell line (HFL). The material was found to be biocompatible, as demonstrated by cytotoxicity assays showing that it did not excessively inhibit fibroblast proliferation. This indicates that the material is likely to be non-toxic in vivo and capable of supporting normal tissue regeneration following injury. Further in vivo studies in a mouse model revealed that the polymer markedly promoted granulation tissue formation, a key stage of wound healing characterised by fibroblast proliferation and neovascularisation. Not only did the material enhance granulation tissue development and angiogenesis, it also significantly accelerated wound closure, reducing wound area by approximately 33% by Day 5 and by over 50% by Day 10 compared to the control group. Histological analyses confirmed reduced local inflammation, increased fibroblast density, substantially greater collagen deposition, early re-epithelialisation, and an absence of bacterial infiltration. Additionally, the sponge exhibited bacteriostatic properties, effectively preventing infection at the wound site. Collectively, these findings suggest that the material is a strong candidate for wound treatment applications [137].

CS-NPs possess unique properties that make them highly attractive for applications in medicine, pharmacy, and technology. These materials are biocompatible, which means that they are well tolerated by the human body without causing toxic reactions. Their biodegradability enables them to naturally break down and be eliminated from the body leaving harmful residues. Chitosan, the base material, has been demonstrated to exhibit a high degree of capacity for binding various active substances, including drugs, enzymes, and genes, thereby facilitating targeted delivery to specific sites [152]. Furthermore, CS-NPs have been shown to be capable of overcoming biological barriers, thus facilitating the controlled release of active agents at the target site. Additionally, encapsulated active substances are protected from premature degradation, modifying their pharmacokinetics [153]. These nanoparticles have also been shown to exhibit antibacterial and anti-inflammatory properties, which makes them beneficial in the treatment of infections and inflammatory conditions [154]. Their small size has been demonstrated to increase interaction with target cell membranes through endocytosis mechanisms [153]. Integration of polyphenols with chitosan nanoparticles in wound healing has been demonstrated to enhance their synergistic effects, with the potential to yield new beneficial properties. This combination has been demonstrated to accelerate tissue regeneration, enhance antibacterial and anti-inflammatory activity, and improve overall protective and reparative characteristics. The utilisation of such solutions has been demonstrated to accelerate the healing process whilst concurrently mitigating the risk of infection and complications, thus establishing them as a promising innovation in advanced wound therapy [151].

In the context of treating diabetic wounds, which are characterised by their recalcitrant nature with regard to healing, the implementation of specialised approaches is imperative. Sun et al. (2020) developed a study of chitosan–gelatin nanoparticles loaded with epigallocatechin gallate (EGCG) and ascorbic acid for the treatment of diabetic wounds [138]. EGCG, a major bioactive compound in green tea, has been shown to exhibit anti-inflammatory, antioxidant, and antibacterial properties [155]. However, its instability, susceptibility to oxidation, and low bioavailability limit its therapeutic performance [156]. The encapsulation of the substance within nanoscale particles has been demonstrated to enhance its stability and efficacy by protecting it from degradation and enhancing its bioavailability [138]. Chitosan’s high positive surface charge ensures nanoparticle stability and active substance transport through various mechanisms [154]. Gelatin, a widely used biopolymer, serves as a biodegradable encapsulating material due to its excellent film-forming ability and oxygen barrier properties [138]. Ascorbic acid, a potent antioxidant, acts as an enzymatic cofactor in processes such as collagen biosynthesis and displays anti-inflammatory effects, thereby supporting immune defence and accelerating wound healing [157]. In vivo studies on diabetic ICR mice demonstrated that EGCG-modified nanoparticles supported wound healing by promoting collagen accumulation and angiogenesis and reducing inflammatory cell infiltration. EV NPS significantly accelerated wound closure, with a markedly higher wound-healing rate than the Model, No-load NPS and EGCG groups from days 2 to 8 (all *p* < 0.001). Furthermore, collagen deposition in the EV NPS group increased significantly compared with the model, no-load NPS and EGCG groups on days 5 and 10 (*p* < 0.001). Immunohistochemical analysis confirmed a substantial reduction in macrophage (F4/80^+^) infiltration in the EV NPS group (*p* < 0.001), indicating more successful inflammation resolution. Reducing inflammation is crucial, as chronic inflammatory responses are known to hinder the healing process and contribute to impaired tissue regeneration [138,158].

Encapsulating quercetin in chitosan nanoparticles overcomes its limitations related to hydrophobicity and poor skin penetration, increasing its potential as a topical therapeutic agent [152]. A study on Wistar rats demonstrated that quercetin-loaded nanoparticles significantly improved wound healing. This was evidenced by reduced TNF-α levels and increased IL-10, VEGF and TGF-β1 expression. On day 21, the 0.03% nanoparticle group had the most mature wounds (11.34 ± 0.21 vs. 10.31 ± 0.25 in controls), superior epithelialisation (3.54 ± 0.08) and collagen fraction (3.63 ± 0.08). The treated wounds also exhibited improved granulation tissue, higher vascular density, reduced inflammatory cell infiltration and more organised collagen architecture. These improvements are the result of the controlled and more precise delivery of quercetin, which leads to stronger biological effects and faster tissue repair [139]. Quercetin-loaded chitosan-lecithin nanoparticles have been shown to enhance water dispersibility, protect the compound from degradation, and increase bioavailability. These nanoparticles have been shown to possess antioxidant and antimicrobial properties, without exerting any cytotoxic effects on L-929 and PBMC cells [159].

A chitosan-based dressing enriched with silver nanoparticles (AgNP) and calendula extract, containing polyphenols, was found to support chronic wound healing by reducing infections and promoting tissue regeneration. AgNPs exhibited potent antimicrobial activity against both Gram-positive and Gram-negative bacteria. They produced inhibition halos measuring up to 0.3 mm for *E. coli* and 0.32 mm for *S. aureus.* The addition of silver resulted in a measurable increase in bactericidal activity, which was absent in the undoped hydrogels. The clinical application of chitosan-based hydrogels enriched with silver nanoparticles (AgNPs) and calendula extract in patients with type II diabetes has demonstrated therapeutic benefits in promoting vascular repair and enhancing the healing of diabetic ulcers. The hydrogel provides a moist wound microenvironment, thereby preserving suitable physiological conditions within the wound bed and the underlying epidermal layers. Moreover, the maintenance of a stable local temperature by the dressing has been demonstrated to facilitate fibrinolytic activity. It has been demonstrated that this process leads to an acceleration in tissue regeneration and a reduction in the likelihood of secondary infections. The findings of clinical trials have confirmed the biocompatibility, safety and high therapeutic value of this composite biomaterial in the management of chronic diabetic wounds. Collectively, these outcomes indicate that the combined use of chitosan, silver nanoparticles and calendula extract has a synergistic therapeutic effect. This surpasses the therapeutic value of each component when used individually, offering an enhanced approach to treating chronic diabetic wounds [28].

Furthermore, the modification of nanoparticles with EGCG has also been demonstrated to have potential in the field of biomedicine. The present study demonstrated that EGCG was capable of inhibiting the growth of various microorganisms such as *E. coli*, *P. aeruginosa*, and *S. aureus*. Chitosan modified with EGCG exhibited a more pronounced antibacterial effect in comparison to EGCG or chitosan administered individually, attributable to elevated ROS generation, which resulted in the impairment of bacterial components. These nanoparticles have also been demonstrated to exhibit superior antioxidant activity through hydrogen atom transfer and reduction mechanisms [160]. The antifungal properties of a combination of chitosan nanoparticles, clotrimazole and grape extract (*Vitis vinifera*) were investigated. The results revealed potent activity against *Candida* and *Aspergillus* species. Although clotrimazole is effective against superficial fungal infections, its limitations, such as toxicity, low bioavailability and emerging resistance, highlight the need for improved delivery systems. It has been demonstrated that grape extract enhances skin regeneration through antioxidant, antimicrobial, antiviral and anticancer mechanisms. Incorporating clotrimazole and grape extract into chitosan nanoparticles significantly improved the bioavailability of the drug and enabled sustained release. The nano-formulation exhibited synergistic antifungal activity, with large inhibition zones of 72 mm against *A. niger* and 74 mm against *C. albicans*, as well as an MIC value of 2 µg/mL for both pathogens. The nanocarrier demonstrated high entrapment efficiency (94.7%), strong antioxidant activity (DPPH = 97.5%), and excellent hemocompatibility (0.3% hemolysis). In vivo studies demonstrated the nano-formulation’s ability to penetrate the skin rapidly, enhance retention and complete tissue repair within seven days, confirming its capability and safety [140]. The study demonstrated that the PCL/chitosan composite nanofiber membrane electro-sprayed with tripolyphosphate–chitosan nanoparticles loaded with curcumin (CURCSNPs) exhibited superior antibacterial, antioxidant, and wound-healing properties in comparison with non-modified fibres. The incorporation of CURCSNPs provided a sustained and gradual release of curcumin over a 16-day period, ensuring continuous therapeutic activity at the wound site. The scaffold demonstrated robust antibacterial activity against methicillin-resistant *S. aureus* (MRSA) and *E. coli*, achieving inhibition rates in excess of 99%. In vitro studies confirmed the enhanced proliferation and viability of human dermal fibroblasts. This was attributed to the hydrophilic surface and bioactive release profile of the material. In vivo, treatment with PCL/CS/CURCSNPs accelerated re-epithelialization, increased collagen deposition, and led to nearly complete wound closure (≈98%) within 15 days in MRSA-infected wounds. Histological analysis revealed well-organised granulation tissue, reduced inflammatory infiltration, and restored epidermal architecture, thus confirming the synergistic role of curcumin’s antioxidant and anti-inflammatory effects with chitosan’s biocompatibility and regenerative potential. Notably, the CURCSNP-modified scaffolds also achieved up to 89% DPPH scavenging activity, indicating a substantial improvement in antioxidant capacity relative to all control groups [141].

The findings summarised in Section 7 demonstrate that enhancements at the mechanistic and molecular level, including augmented antioxidant activity, modulation of inflammatory signalling, sustained antimicrobial action, increased angiogenic potential, and enhanced regulation of cellular proliferation, are pivotal in restoring the fundamental regenerative processes necessary for effective wound repair. These mechanistic enhancements have been demonstrated to support more efficient granulation tissue development, accelerate re-epithelialisation, and promote better organisation of the extracellular matrix. Collectively, these effects have been shown to strengthen the wound bed and reduce the likelihood of secondary complications. The biomaterials described in this section have been shown to stabilise these early healing processes, thereby minimising infection, excessive exudation, and delays in tissue remodelling. The combination of these meticulously planned enhancements has been shown to enhance the probability of achieving clinically optimal outcomes, including accelerated, stable, and complication-free wound closure.

## 8. Conclusions

Chronic wounds, particularly those associated with diabetes, persist as a substantial clinical and economic challenge due to prolonged healing times, high susceptibility to infection, and the limited effectiveness of conventional treatment approaches. The persistent absence of satisfactory therapeutic outcomes underscores the pressing need for innovative, biologically informed strategies to facilitate tissue repair and regeneration.

The integration of chitosan nanostructures (CS-NSs) with polyphenols is a promising avenue for the development of advanced wound healing systems. This approach addresses key limitations related to the poor stability and bioavailability of polyphenols, allowing for their controlled and localised release at the wound site. The inherent physicochemical properties of CS-NSs, such as biocompatibility, biodegradability, and antimicrobial activity, further contribute to their potential as multifunctional carriers.

The encapsulation of bioactive molecules, including EGCG, quercetin, and curcumin, within CS-NSs has been demonstrated to assist in the preservation of their inherent biological activity, whilst concomitantly amplifying their antioxidant, anti-inflammatory, and regenerative effects. Furthermore, the incorporation of supplementary components, such as silver nanoparticles, gelatin, or plant-derived extracts, has been demonstrated to yield synergistic enhancements in antimicrobial protection and tissue regeneration.

While these developments significantly enhance the therapeutic potential of the systems combining chitosan and polyphenols, it is unlikely that they alone will fully resolve the complex biological and clinical challenges associated with chronic wound management. Future progress will depend on a deeper mechanistic understanding of how chitosan-based nanostructures interact with biological tissues and how these interactions can be fine-tuned through rational material design. The present review aims to bridge the gap between material engineering and biological mechanisms by providing a comprehensive, mechanistic overview of chitosan–polyphenol nanocomposites and their potential integration with metal nanoparticles. Ultimately, the review outlines promising directions for future wound healing research.

In light of current evidence, nanostructure-based chitosan–polyphenol systems demonstrate measurable progress in addressing key barriers to chronic wound healing, including infection control, reduction of oxidative stress, and stimulation of angiogenesis. While they do not yet constitute a complete therapeutic solution, they provide a solid foundation for the rational design of next-generation dressings and bioactive compound delivery systems.

Future research should concentrate on establishing translational relationships between material properties and outcomes observed in in vitro, in vivo, and clinical studies. It is imperative to ascertain whether enhancements such as enhanced angiogenesis, improved inflammatory control, augmented antimicrobial effects, and more organised extracellular matrix formation are adequate to expedite wound closure and mitigate complication rates. It is imperative to demonstrate that these mechanistic effects reliably translate into faster, more stable, and complication-free healing. This will allow for the identification of the combinations of chitosan modifications, metal nanoparticles, and polyphenols that offer the most clinically relevant balance of safety, durability, and biological performance.

This review may serve as a reference point in the identification and optimisation of biomaterials with therapeutic potential in wound healing, including inflammation reduction, cell migration support, and tissue remodelling. Further quantitative studies and controlled clinical evaluations are essential to confirm their practical applicability and long-term therapeutic benefits.

## Data Availability

No new data were created or analyzed in this study. Data sharing is not applicable to this article.

## Abbreviations

AgNPs	silver nanoparticles
AgNPs@Chi	chitosan-based matrices containing silver nanoparticles
Akt	protein kinase B
APA-AuNPs	6-aminopenicillanic acid–coated gold nanoparticles
AuNPs	gold nanoparticles
bFGF	basic fibroblast growth factor
CA	carboxylic acid
CCS	chitosan sponge
CD31	cluster of differentiation 31
CD34	cluster of differentiation 34
ChiZn	chitosan–zinc complexes
CMCS	carboxymethyl chitosan
CS-AgNPs	chitosan–PVA–silver nanoparticles
CS-NPs	chitosan nanoparticles
CuNPs	copper nanoparticles
DHQ	dihydroquercetin
ECM	extracellular matrix
EGCG	epigallocatechin gallate
EV NPS	extracellular vesicle nanoparticles
FDA	Food and Drug Administration
FGF-2	fibroblast growth factor-2
GAGs	glycosaminoglycans
GO	graphene oxide
IL-10	interleukin-10
IL-1β	interleukin-1 beta
IL-6	interleukin-6
IONPs	iron oxide nanoparticles
KGF	keratinocyte growth factor
Ki67	cell proliferation marker
LL37	lactoferricin-like peptide 37
MACS	hemostatic sponges
MAPK	mitogen-activated protein kinase
MDR	multidrug resistance
MMP-3	matrix metalloproteinase-3
MNPs	metal-based nanoparticles
mRNA	messenger ribonucleic acid
MRSA	methicillin-resistant *Staphylococcus aureus*
MTT	3-(4,5-dimethylthiazol-2-yl)-2,5-diphenyltetrazolium bromide
NFs	nanofibres
NF-κB	nuclear factor kappa-light-chain-enhancer of activated B cell
NLCs	nanostructured lipid carriers
NPs	nanoparticles
p53	tumor protein p53
PAA	polyacrylic acid
PCL	poly-ε-caprolactone
PEG	polyethylene glycol
PGA	polyglycolic acid
PI3K	phosphoinositide 3-kinase
PU	polyurethane
PVA	polyvinyl alcohol
PVP	polyvinylpyrrolidone
Q	quercetin
QSAR	quantitative structure–activity relationship
REO	rosemary essential oil
ROS	reactive oxygen species
SLNs	solid lipid nanoparticles
SOD	superoxide dismutase
TA	tannic acid
TGF-β1	transforming growth factor beta 1
TNF-α	tumor necrosis factor alpha
UsAuNPs	ultrasmall gold nanoparticles
VEGF	vascular endothelial growth factor
ZnO-NPs	zinc oxide nanoparticles
